# Supplementary material for: A Systematic Review and Meta-analysis Protocol on Depressive Symptoms Among Medical Students in South Asia Using Patient-reported Validated Assessment Tools: Prevalence and Associated Factors
Source: PLoS One. 2025 Aug 26;20(8):e0331033. doi: 10.1371/journal.pone.0331033 (PMC12380302; doi:10.1371/journal.pone.0331033)
Supplement: S2 Table — Search strategy for PubMed, PsycINFO, Scopus, CINAHL, EMBASE. (DOCX) [file pone.0331033.s002.docx]

**Supplementary Table (S1): Search strategy for PubMed, PsycINFO, Scopus, CINAHL, EMBASE**

- **Strategy for PubMed (Medline Database)**

| **1.** | (((Depression[Title/Abstract]) OR (Depressive disorder[Title/Abstract])) OR (Mood disorder[Title/Abstract])) OR (Affective disorder[Title/Abstract]) |
| --- | --- |
| **2.** | ("medical students"[MeSH] OR "intern doctors"[MeSH] OR "undergraduate medical students"[MeSH] OR "postgraduate medical students"[MeSH] ) |
| **3.** | ((((((((((Epidemiology[Title]) OR (Prevalence[Title])) OR (Trends[Title])) OR (Risk factors[Title])) OR (Associated factors[Title])) OR (Determinants[Title])) OR (Contributing factors[Title])) OR (Protective factors[Title])) OR (Aggravating factors[Title])) OR (Modifiable factors[Title])) OR (Non-modifiable factors[Title]) |
| **4.** | ((((((((((((((((((((South Asia[MeSH Terms]) OR (Southern Asia[MeSH Terms])) OR (South Asia*[MeSH Terms])) OR (Afghanistan*[MeSH Terms])) OR (Afghan[MeSH Terms])) OR (Bangladesh*[MeSH Terms])) OR (Bangladeshi[MeSH Terms])) OR (India*[MeSH Terms])) OR (Indian[MeSH Terms])) OR (Bhutan*[MeSH Terms])) OR (Bhutanese[MeSH Terms])) OR (Sri Lanka*[MeSH Terms])) OR (Sri Lankan[MeSH Terms])) OR (Maldives*[MeSH Terms])) OR (Maldivian[MeSH Terms])) OR (Nepal*[MeSH Terms])) OR (Nepalese[MeSH Terms])) OR (Pakistan*[MeSH Terms])) OR (Pakistani[MeSH Terms])) OR (Lanka*[MeSH Terms])) OR (Ceylon*[MeSH Terms]) |
| **5.** | #1 AND #2 AND #3 AND #4 |
| **6.** | Filters: Free full text, Full text |

- **Search Strategy for PsycINFO**

| **1.** | (DE "Depression" OR DE "Major Depression" OR DE "Depressive Disorders" OR DE "Mood Disorders" OR DE "Affective Disorders" OR TI("Depression" OR "Depressive disorder" OR "Mood disorder" OR "Affective disorder") OR AB("Depression" OR "Depressive disorder" OR "Mood disorder" OR "Affective disorder")) |
| --- | --- |
| **2.** | (DE "Medical Students" OR DE "Interns (Medical)" OR TI("medical students" OR "intern doctors" OR "undergraduate medical students" OR "postgraduate medical students") OR AB("medical students" OR "intern doctors" OR "undergraduate medical students" OR "postgraduate medical students")) |
| **3.** | (TI("Epidemiology" OR "Prevalence" OR "Trends" OR "Risk factors" OR "Associated factors" OR "Determinants" OR "Contributing factors" OR "Protective factors" OR "Aggravating factors" OR "Modifiable factors" OR "Non-modifiable factors")) |
| **4.** | (TI("South Asia*" OR "Southern Asia" OR "Afghanistan*" OR "Afghan" OR "Bangladesh*" OR "Bangladeshi" OR "India*" OR "Indian" OR "Bhutan*" OR "Bhutanese" OR "Sri Lanka*" OR "Sri Lankan" OR "Maldives*" OR "Maldivian" OR "Nepal" OR "Nepalese" OR "Pakistan*" OR "Pakistani" OR "Lanka" OR "Ceylon")  OR AB("South Asia" OR "Southern Asia" OR "Afghanistan" OR "Afghan" OR "Bangladesh" OR "Bangladeshi" OR "India" OR "Indian" OR "Bhutan" OR "Bhutanese" OR "Sri Lanka" OR "Sri Lankan" OR "Maldives" OR "Maldivian" OR "Nepal" OR "Nepalese" OR "Pakistan" OR "Pakistani" OR "Lanka" OR "Ceylon")) |
| **5.** | #1 AND #2 AND #3 AND #4 |

- **Search Strategy for Scopus via Elsevier**

| **1.** | (TITLE-ABS("Depression") OR TITLE-ABS("Depressive disorder") OR TITLE-ABS("Mood disorder") OR TITLE-ABS("Affective disorder")) |
| --- | --- |
| **2.** | (TITLE-ABS("medical students") OR TITLE-ABS("intern doctors") OR TITLE-ABS("undergraduate medical students") OR TITLE-ABS("postgraduate medical students")) |
| **3.** | (TITLE("Epidemiology") OR TITLE("Prevalence") OR TITLE("Trends") OR TITLE("Risk factors") OR TITLE("Associated factors") OR TITLE("Determinants") OR TITLE("Contributing factors") OR TITLE("Protective factors") OR TITLE("Aggravating factors") OR TITLE("Modifiable factors") OR TITLE("Non-modifiable factors")) |
| **4.** | (TITLE-ABS("South Asia") OR TITLE-ABS("Southern Asia*") OR TITLE-ABS("Afghanistan*") OR TITLE-ABS("Afghan") OR TITLE-ABS("Bangladesh*") OR TITLE-ABS("Bangladeshi") OR TITLE-ABS("India*") OR TITLE-ABS("Indian") OR TITLE-ABS("Bhutan*") OR TITLE-ABS("Bhutanese") OR TITLE-ABS("Sri Lanka") OR TITLE-ABS("Sri Lankan*") OR TITLE-ABS("Maldives*") OR TITLE-ABS("Maldivian") OR TITLE-ABS("Nepal*") OR TITLE-ABS("Nepalese") OR TITLE-ABS("Pakistan*") OR TITLE-ABS("Pakistani") OR TITLE-ABS("Lanka") OR TITLE-ABS("Ceylon")) |
| **5.** | #1 AND #2 AND #3 AND #4 |
| **6.** | Filters: Free full text |

- **Search Strategy for CINAHL**

| **1.** | (TI "Depression" OR AB "Depression" OR TI "Depressive disorder" OR AB "Depressive disorder" OR TI "Mood disorder" OR AB "Mood disorder" OR TI "Affective disorder" OR AB "Affective disorder") |
| --- | --- |
| **2.** | (MH "Students, Medical" OR MH "Internship and Residency" OR TI "medical students" OR AB "medical students" OR TI "intern doctors" OR AB "intern doctors" OR TI "undergraduate medical students" OR AB "undergraduate medical students" OR TI "postgraduate medical students" OR AB "postgraduate medical students") |
| **3.** | (TI "Epidemiology" OR TI "Prevalence" OR TI "Trends" OR TI "Risk factors" OR TI "Associated factors" OR TI "Determinants" OR TI "Contributing factors" OR TI "Protective factors" OR TI "Aggravating factors" OR TI "Modifiable factors" OR TI "Non-modifiable factors") |
| **4.** | (MH "Asia, Southern" OR TI "South Asia*" OR AB "South Asia" OR TI "Southern Asia" OR AB "Southern Asia" OR TI "Afghanistan*" OR AB "Afghanistani" OR TI "Bangladesh*" OR AB "Bangladeshi" OR TI "India*" OR AB "Indian" OR TI "Bhutan" OR AB "Bhutan" OR TI "Sri Lankan" OR AB "Sri Lanka*" OR TI "Maldives*" OR AB "Maldivian*" OR TI "Nepalese" OR AB "Nepal*" OR TI "Pakistan*" OR AB "Pakistani" OR TI "Ceylon" OR AB "Ceylon") |
| **5.** | #1 AND #2 AND #3 AND #4 |

- **Search Strategy for EMBASE**

| **1.** | ('depression'/exp OR 'depressive disorder'/exp OR 'mood disorder'/exp OR 'affective disorder'/exp OR title:depression OR abstract:depression OR title:'depressive disorder' OR abstract:'depressive disorder' OR title:'mood disorder' OR abstract:'mood disorder' OR title:'affective disorder' OR abstract:'affective disorder') |
| --- | --- |
| **2.** | ('medical student'/exp OR 'internship'/exp OR title:'medical student' OR abstract:'medical student' OR title:'intern doctor' OR abstract:'intern doctor' OR title:'undergraduate medical student' OR abstract:'undergraduate medical student' OR title:'postgraduate medical student' OR abstract:'postgraduate medical student') |
| **3.** | (title:epidemiology OR title:prevalence OR title:trends OR title:'risk factors' OR title:'associated factors' OR title:determinants OR title:'contributing factors' OR title:'protective factors' OR title:'aggravating factors' OR title:'modifiable factors' OR title:'non-modifiable factors') |
| **4.** | ('south asia*'/exp OR 'southern asia'/exp OR 'afghanistan*'/exp OR 'bangladesh*'/exp OR 'india*'/exp OR 'bhutan*'/exp OR 'sri lanka*'/exp OR 'maldives*'/exp OR 'nepal*'/exp OR 'pakistan*'/exp OR title:'south asia' OR abstract:'south asia' OR title:'afghanistan' OR abstract:'afghanistan' OR title:'bangladesh' OR abstract:'bangladesh' OR title:'india' OR abstract:'india' OR title:'bhutan' OR abstract:'bhutan' OR title:'sri lanka' OR abstract:'sri lanka' OR title:'maldives' OR abstract:'maldives' OR title:'nepal' OR abstract:'nepal' OR title:'pakistan' OR abstract:'pakistan' OR title:'ceylon' OR abstract:'ceylon') |
| **5.** | #1 AND #2 AND #3 AND #4 |
