# Supplementary material for: A Systematic Review and Meta-analysis Protocol on Depressive Symptoms Among Medical Students in South Asia Using Patient-reported Validated Assessment Tools: Prevalence and Associated Factors
Source: PLoS One. 2025 Aug 26;20(8):e0331033. doi: 10.1371/journal.pone.0331033 (PMC12380302; doi:10.1371/journal.pone.0331033)
Supplement: Attachment — Submitted filename: Response to Reviewers.docx [file pone.0331033.s004.docx]

Dear Editor,

**Jiawen Deng**

PLOS One.

The authors would like to express their heartfelt gratitude for the opportunity to submit the revised version of the manuscript to all the respected reviewers and the editors. Having received the mentioned suggestions made by the editor and reviewers had helped significantly to improve the overall content at all.

Our point-by-point responses to the editor and reviewers’ comments can be found below accordingly. The suggested changes have been incorporated in the revised version of the manuscript and are attached with a track change version. We tried our best to address all the comments raised by the authors and hope that the paper is now acceptable for publication in PLOS One.

Thank you very much, and we are looking forward to hearing from you soon.

On behalf of all the authors,

**Dr. Mantaka Rahman (Corresponding author)**

25^th^ June, 2025.

Dhaka, Bangladesh.

**RESPONSES TO THE ADDITIONAL EDITOR**

**Additional Editors Comment:**

1. **Editor’s Comment:** Authors' claims to be the first comprehensive synthesis are inaccurate. In fact, the authors cited several studies themselves that already pooled prevalences and risk factors across South Asian nations. Please justify the novelty and necessity of the proposed review.

***Authors’ response:*** Thank you for the valuable insight. We acknowledge that previous reviews have pooled prevalence and associated factors of depression among South Asian medical students. However, our review adds novelty by focusing specifically on studies that use validated patient-reported assessment tools (e.g., PHQ-9, BDI, HADS), allowing for a more standardized and comparable synthesis. In addition, our aim to address gaps in recent data across countries, particularly from low- and middle-income settings. To capture recent data, explore regional heterogeneity across countries, and include above mentioned grey literature sources often excluded from prior syntheses. These enhancements are intended to strengthen the evidence synthesis and provide more actionable insights for region-specific mental health interventions in this context.

2. **Editor’s Comment:** The term "associated factors" is ill-defined. Please discuss how these factors will be categorized or specifically which factors will be assessed. Systematic reviews need to have a concrete research question; "associated factors" is too vague. ***Authors’ response:*** Thank you for this concern. We agree that “associated factors” requires greater specificity based on the outcome of interest of the review. In our revised manuscript, we have clarified that associated factors refer to demographics (e.g., age, gender), academic (e.g., exam stress, study hours, academic performance), psychosocial (e.g., sleep quality, social support), institutional (access to mental health service, bullying, accommodation), and behavioral (e.g., screen time, substance use). These categories were supported by existing literature and other related categories (if available) will also be used to systematically extract as the associated factors. A narrative synthesis approach will be followed to extract the factors from the included studies to answer the research question.

3. **Editor’s Comment:** How exactly will the NOS be modified? Why are only medium and good quality studies included? RoB ratings can be subjective and there is a risk of leaving out relevant studies. Usually low risk studies are examined via sensitivity/subgroup analyses by RoB and rarely are they explicitly excluded.

***Authors’ response:*** Thank you for the good observation. Although we previously planned to included only medium and good quality studies which may reduce the generalizability of the study and inconsistent result. Thus, as per your valuable suggestion in the revised version we have decided to include all studies (good, medium and poor) regardless RoB and further examine via sensitivity/subgroup analyses to maintain the methodological rigor.

4. **Editor’s Comment:** How will the effects of "associated factors" be assessed? Will there be any pooling or quantitative synthesis? If so, will these be assessed as odds ratios, meta-regressions, or something else?

***Authors’ response:*** Thank you for your comment. We only plan to conduct a narrative synthesis of factors to identify and summarize the associated factors of depression among medical students, as reported in the included studies. Due to expected variability in study designs, measurement tools, and reported effect sizes, a quantitative synthesis (e.g., meta-analysis) of associated factors may not be feasible (Campbell et al., 2018). For narrative synthesis, the effects of associated factors will be assessed systematically by summarizing the reported associations across reported studies.

5. **Editor’s Comment:** Please name specific grey literature sources that will be searched. ***Authors’ response:*** The authors plan to find specific grey literature sources including ProQuest, MedRxiv, Google Scholar, Government reports, and regional online journals such as. BanglaJOL (Bangladesh), NepJOL (Nepal), PakJOL (Pakistan), SLJOL (SriLanka), Shodhganga (India). The following gray literature sources were incorporated in the revised version.

6. **Editor’s Comment:** In general, there is language issues. There are long rambling sentences which are hard to read.

***Authors’ response:*** Thank you for the observation. We apologies for the inconsistency in the language issues. None of the first authors are native English speaker. However, in the revised version we tried to accommodate the issues and long rambling sentences are made a simple one for clarity.

7. **Editor’s Comment:** Please review your introduction and discussions, and make sure that your statements regarding mental health stigma and systemic challenges are backed by academic citations.

***Authors’ response:*** Thank you for the observation. We have carefully rechecked the following aspects and necessary academic citations were incorporated accordingly in the above mention sections in the revised version.

8. **Editor’s Comment:** Certain parts of the manuscript seems to have been copy pasted,? from somewhere else. The part on heterogeneity is mentioned twice in the manuscript, for instance.

***Authors’ response:*** We kindly apologizes for the inconvenience. The authors tried to explain heterogeneity briefly in the second section. However, for more clarity the duplicate portion of heterogeneity is revised and corrected. The authors sincerely checked thoroughly.

9. **Editor’s Comment:** Will any transformation be applied to the prevalence values prior to pooling? Please clarify statistical plan.

***Authors’ response:*** Thank you for your nice statistical query. Yes, we do have plan to apply the Freeman–Tukey double arcsine transformation to stabilize variance and reduce the impact of extreme prevalence values prior to pooling (for non-normally distributed data). After that, as mentioned, a random-effects model will be used to estimate the pooled prevalence across studies.

10. **Editor’s Comment:** There is no GRADE framework available for prevalence meta-analyses. Please remove this.

***Authors’ response:*** We completely agree with your comment and the following section has been removed in the revised version.

11. **Editor’s Comment:** Please remove all figures. You do not need placeholder figures nor do you need a figure for your introduction. The last figure (network plot) seems to be for illustrative purposes only and serve no real purpose.

***Authors’ response:*** Thank you for your valuable concern. We comply with your suggestions and agree that we do not need figure for the introduction section. However, the figures including the network plot were used for visualization of the process like PRISMA, screening process, and overall prevalence globally at a glance. In respect to your suggestions we have removed all the figures from the main section rather added them as a supplementary file which will be accessible for anyone.

12. **Editor’s Comment:** You mention time cutoff for included articles being March 2025 in one section of the manuscript but May 2025 in other sections. Please explain this discrepancy.

***Authors’ response:*** We apologizes for the inconsistency. The cut-off timeframe was up-to 1^st^ May 2025 and thus revised accordingly throughout the sections.

Thank you for your consideration.

**RESPONSES TO REVIEWER 1**

1. **Reviewer’s comment:** Understanding the prevalence of depression and associated risk factors is essential to guide targeted interventions and evidence-based policy reforms that support the mental well-being of future healthcare professionals. By systematically synthesizing data from observational studies, this review will provide a comprehensive synthesis of depression prevalence and its correlates among medical students in the South Asian region, laying the groundwork for preventive strategies and improved mental healthcare practices.

**Authors’ response:** Thank you for your positive feedback and the authors really appreciate your valuable comments and concerns. Further comments are addressed point by point to improve the overall merit and reliability of the manuscript.

**RESPONSES TO REVIEWER 2**

1. **Reviewer’s comment:** I would like to express my sincere appreciation for the opportunity to review this valuable and comprehensive work. The manuscript presents an insightful and systematic meta-analysis study protocol for depression among medical students in South Asia, with a strong methodological framework and a high quality of included studies.

**Authors’ response:** Thank you for your valuable insight and further comments are addressed point by point according in the revised version of the manuscript.

2. **Reviewer’s comment:** The objective is clearly defined, and the author used PICO (Population, Intervention, Comparator, Outcome).

**Authors’ response:** We appreciate and thank for the valuable comment of the reviewer.

3. **Reviewer’s comment:** As a depression case, what are the standard criteria used among different studies to give a standard diagnosis of depression among medical students in the South Asian region? Should be stated.

**Authors’ response:** Thank you for your insightful comment. As stated in the eligibility, studies will be included if they use standardized and validated patient-reported depression assessment tools such as the PHQ-9, BDI, and HADS. However, based on cut-off scores recommended by the respective instruments (e.g., PHQ-9 ≥10 for moderate depression), based on DSM-IV diagnostic criteria, will be used for level of depression categorization. To ensure clarity and transparency, we have stated and revised the manuscript to state that variations in measurement tools and diagnostic thresholds will be examined through subgroup and sensitivity analyses.

4. **Reviewer’s comment:** The author didn’t define depression cases in different studies.

**Authors’ response:** We acknowledge the comment. As previously mentioned, the depression cased across different studies will be defined using the patient-reported standardized and psychometrically validated tools (e.g., PHQ-9, BDI, HADS) following DSM-IV criteria. This way of defining depression cases in different studies is incorporated in the revised version accordingly.

5. **Reviewer’s comment:** Regarding I² categorized as low (≤25%), moderate (50%), or high (≥75%), what about the range from 25% to 50%?

**Authors’ response:** We apologies for the inconsistence in the categorization. In the revised version we clarified the ranges accordingly and categorized as low heterogeneity (0-25%), moderate heterogeneity (25-75%), or high heterogeneity (>75%).

6. **Reviewer’s comment:** For depression measuring tools, what is the difference between self-reported and patient-reported?

**Authors’ response:** Thank you for this valuable observation. We acknowledge that the terms self-reported and patient-reported were used interchangeably in the initial draft, which may have led to some confusion. We truly appreciate some inconsistence occurred in the manuscript. However, in the revised version a single terminology, patient-reported, was used throughout the manuscript to maintain consistency.

7. **Reviewer’s comment:** Although the topic is clinically or scientifically significant, any justification for conducting this study since many other similar meta-analyses were conducted globally?

**Authors’ response:** Thank you for your comment. While several global meta-analyses have assessed depression among medical students, region-specific data is crucial to compare the vulnerability between low-and-middle-income countries (LMICs) and high-income countries (HICs), especially considering ~60.69% of medical students are from LMICs (Akhter et al., 2020). In addition, there are sporadic studies in South Asia and lack regional synthesis; therefore, generating a pooled prevalence rate and investigating related factors in this region will provide potential evidence to inform further mental health strategical planning and address the growing disease burden in this context.

8. **Reviewer’s comment:** The study's rationale didn’t justify the need for a new review…

**Authors’ response:** Thank you for the observation. Although we have clarified the rationale. However, according to the concern of the reviewer we have revised and tried to justify the need for a new review and establish the rationale of the study accordingly.

9. **Reviewer’s comment:** Inclusion and exclusion criteria are clearly specified.

**Authors’ response:** We appreciate the valuable comments and insights of the reviewer.

10. **Reviewer’s comment:** The author should elaborate more in the discussion.

**Authors’ response:** According to the reviewer suggestion the discussion section has been elaborated with necessary revenant information accordingly in the revised manuscript.

**RESPONSES TO REVIEWER 3**

1. **Reviewer’s comment:** This protocol presents a timely and well-structured plan for a systematic review and meta-analysis addressing the prevalence and associated factors of depression among medical students in South Asia. The rationale is strong and grounded in regional disparities in mental health research, with a clear and relevant research question formulated using the PICO framework.

The proposed methodology is rigorous, employing best practices such as PRISMA, MOOSE, and NOS guidelines, and registration in PROSPERO. The inclusion of subgroup analysis and sensitivity testing further adds to the protocol’s analytical strength.

**Authors’ response:** Thank you for your positive feedback and commenting on the overall methodology and justification of the proposed review. We appreciate all your further suggestions and addressed point by point accordingly in the revised version to improve the overall quality of the manuscript.

2. **Reviewer’s comment:** (**Strengths)**

Comprehensive search strategy, including grey literature and citation chaining. Transparent risk of bias assessment and use of validated scales (e.g., PHQ-9, BDI).

Use of standard software (R, meta package) and appropriate statistical models.

Clear data availability and ethics statements.

**Authors’ response:** Thank you for your valuable comment regarding the strength of the manuscript. Further comments and suggestions are addressed accordingly in the revised version.

3. **Reviewer’s comment:** Timeline Clarification: The projected timeline (completion in mid-2026) seems extended relative to typical protocol expectations. Consider justifying or adjusting this to reflect a more standard 12–18 month execution window.

**Authors’ response:** Thank you for your concern. We appreciate your comment on the previous timeline calculation. The 12-18 month execution window is best fit for the study and thus we adjusted this timeframe and revised accordingly. We assure you the standard timeline along with milestones will be followed appropriately mentioned in the protocol.

4. **Reviewer’s comment:** Subgroup Exclusions: Excluding medical students with comorbid physical health conditions may omit relevant populations. Please clarify the rationale or consider including such data with subgroup analysis.

**Authors’ response:** We completely agree with the reviewer regarding the point. Excluding the students with comorbid physical health conditions may affect the overall population thus we will include such group and represent the data with a subgroup analysis.

5. **Reviewer’s comment:** Typographical Edits: Minor inconsistencies in English usage (e.g., “analysed” vs. “analyzed”) and repeated use of placeholders like “XXXX” in figures. A proofreading pass is recommended to ensure consistency and polish.

**Authors’ response:** We appreciate your insight regarding the minor inconsistencies’ typographical edits throughout the manuscript and thus manuscript’s proofreading was rechecked carefully by the authors and addressed accordingly for the overall consistency. In addition, the inconsistency in figure was corrected also.

6. **Reviewer’s comment:** Overall, the protocol is well-prepared and likely to yield meaningful and actionable insights for medical educators, policymakers, and researchers across South Asia. I recommend acceptance following minor revisions.

**Authors’ response:** Thank you for your feedback. We appreciate your valuable insight.
