# Supplementary material for: A Systematic Review and Meta-analysis Protocol on Depressive Symptoms Among Medical Students in South Asia Using Patient-reported Validated Assessment Tools: Prevalence and Associated Factors
Source: PLoS One. 2025 Aug 26;20(8):e0331033. doi: 10.1371/journal.pone.0331033 (PMC12380302; doi:10.1371/journal.pone.0331033)
Supplement: Attachment — Submitted filename: Response_to_Reviewers_auresp_2.docx [file pone.0331033.s005.docx]

23^th^ July, 2025.

**Jiawen Deng**

Academic Editor

PLOS One

Dear Editor,

The authors sincerely thank the editor, additional editor, and the respected reviewer for providing valuable concern on our manuscript. We greatly appreciate the opportunity to submit this revised version.

The insightful comments by the additional editor have been extremely helpful in enhancing the overall quality and clarity of the protocol manuscript. We have carefully addressed each point raised, and our detailed, point-by-point responses are attached below.

All the suggested changes and queries have been incorporated into the revised manuscript, which is submitted herewith along with a tracked changes version for your reference. We believe that the manuscript has improved substantially and hope it now meets the standards for publication in PLOS One.

Thank you once again for your kind consideration. We look forward to your next feedback.

On behalf of all the authors,

**Dr. Mantaka Rahman** _(Corresponding author)_

Dhaka, Bangladesh.

**RESPONSES TO THE ADDITIONAL EDITOR**

**Additional Editor Comment:**

1. **Editor’s Comment:** It is probably inappropriate to say that the review itself will follow PRISMA-P. The review itself should follow PRISMA, but the protocol should be reported following PRISMA-P.

***Authors’ response:*** Thank you for your comment. We agree with your concern that the protocol for this review adheres to PRISMA-P while the main reporting of the review will be following PRISMA. The related section in abstract, study design, and strength and limitation section has been revised accordingly ***(Page number 2, 6,15).***

2. **Editor’s Comment:** At this stage I would like for the authors to lock in their information sources. Instead of saying "from a wide range of reputable sources, INCLUDING..." Simply list all databases that will be used.

***Authors’ response:*** As per the editor’s suggestion we have clearly defined and locked the number of information sources we will use. In the information sources section, we have specified both the exact number and names of the electronic database sources to for improved clarity in the revised version ***(Page number 7-8).***

3. **Editor’s Comment:** The condition section is still not clear. Again, please list all standardized and validated tools that you are seeking in the included studies. Please also include threshold values as these tools are validated at a certain threshold level.

For associated factors, please provide the full list instead of giving examples of each category. The review methodology must be concrete in this regard.

***Authors’ response:*** Thank you for your valuable suggestion. We have tried to clearly clarify the condition section by adding the threshold/cut-off value of each validated assessment tools including PHQ-9 (≥10), BDI/BDI-II (≥14), BDI-SF (≥8), HADS (≥8), CES-D (≥16), ZSDS (≥50), DASS-21/DASS-42 (≥10) which we are seeking in the included studies in the revised manuscript ***(Page number: 9-10).***

Regarding the associated factors, we have now provided the full list of associated factors identified in the review, categorized by 1) socio-demographic, 2) psychosocial, 3) academia, and 4) lifestyle domains. The section has also been reflected in the updated version ***(Page number: 9-10).***

4. **Editor’s Comment:** Please explain the difference between MBBS and undergraduate medical students.

***Authors’ response:*** Thank you for raising the point. In our context, MBBS/BDS refers to individuals who have already completed their medical or dental degree programs and are considered medical graduates or MBBS doctors in countries such as Bangladesh, India, and Nepal. In contrast, undergraduate medical students refer to those currently enrolled in the medical program (typically from 1^st^ to 5^th^ academic year) who have not yet completed their degree or obtained a license to practice. For more clarity, we have revised the terminology in the manuscript accordingly ***(Page number: 9).***

5. **Editor’s Comment:** Please avoid the term "depression" in your protocol, as validated scales (such as PHQ-9) only screens for depressive SYMPTOMS, and they do not provide a formal diagnosis of depression.

***Authors’ response:*** Thank you for the clarification. We acknowledge your concern that tools like the PHQ-9, BDI screen for depressive symptoms rather than providing a clinical diagnosis. However, we used the

term "depression" in line with how it will be reported in the included studies, where it is commonly referred to elevated depressive symptoms as "depression" in many literatures (DOI: 10.1001/jama.2016.17324, DOI: 10.1371/journal.pone.0221432, DOI: 10.1186/s12888-017-1382-3). But, based on your suggestion, we have revised the entire manuscript, title, and where appropriate to clarify that our analysis reflects depressive symptoms, not clinical diagnoses of Depression.

6. **Editor’s Comment:** Please provide the full list of demographic information being extracted.

***Authors’ response:*** Thank you for your insightful comment. We will extract the following demographic information from each included study (age, sex, ethnicity, academic year, academic level, institution, geographic location, familial history, marital status, socio-economic status). Additional relevant demographic variables reported in the studies will also be recorded where available. In the revised version we have clarified each details of the tentative information being extracted ***(Page number: 11).***

7. **Editor’s Comment:** Authors mentioned "The main focus will be on extracting...any reported levels of depression severity (e.g., mild, moderate, severe) based on DSM-IV criteria." Please confirm that you are aiming to extract severity based on DSM instead of different threshold levels in the validated tools. It is unclear how severity can be extracted based on DSM.

***Authors’ response:*** Thank you for raising the observation. We agree with your concern and apologize for the confusion. We confirm that we will extract depression severity based on threshold / cut-off scores of validated screening tools (e.g., PHQ-9, BDI-II, DASS-21), as reported by the original included studies in the review, not based on DSM-IV criteria. The statement has been corrected accordingly in the revised manuscript ***(Page number: 11).***

8. **Editor’s Comment:** What kind of modification is being done to NOS? Also note that different versions of the NOS exists for different study types; please specify which one will be used.

***Authors’ response:*** Thank you for your valuable comment. We will assess study quality using the Newcastle-Ottawa Scale (NOS) adapted for cross-sectional studies as the majority of included studies are expected to be observational and cross-sectional in nature. Minor modifications include defining adequate sample size (≥150), representativeness (random/multi-institutional sampling, inclusion of both genders and academic years), and use of validated outcome assessment tools. The modified tool will be provided in the appendix section of the main review and will be used based on study design. In the revised version we have clarified it properly ***(Page number: 12).***

9. **Editor’s Comment:** There is still a redundant section on "Strategy for data synthesis", which curiously contains a copy-pasted version of the database search methodology.

Please very carefully review your manuscript to ensure that these types of errors are not included.

***Authors’ response:*** We kindly apologizes for the errors. The section "Strategy for data synthesis", has been revised accordingly and the redundancy has been removed in the revised version ***(Page number: 13).***

10. **Editor’s Comment:** Please remove the section on status and timeline of the study. This is not typically included in systematic review protocols.

***Authors’ response:*** According to the suggestion of the editor, the section pertaining “The status and timeline of the study” has been removed in the revised manuscript (***Page number: 13)***.

11. **Editor’s Comment:** The incorrect checklist is attached. You should attach the PRISMA-P checklist, not the PRISMA checklist (this one is reserved for your final review).

***Authors’ response:*** We agree with your concern. In the revised version we have added the PRISMA-P checklist accordingly.

12. **Editor’s Comment:** Please review whether your figures are necessary. Figures 1 and 2 are not needed as they contain no data. Likewise, Figure 3 does not contain any information and it is unclear what the graph represents or what readers are supposed to draw from it. Figure 4 contains a summary of data from other studies and this is not typically seen in protocol publications.

***Authors’ response:*** Thank you for your valuable comment. We agree with your concern and have removed Figures 1, 2, and 3 accordingly. However, we believe the global summary visualization in Figure 4 offers valuable context by providing a concise overview of global statistics. We think it can be helpful for readers. To have quick overview. Therefore, we have retained it as Figure 1 in the discussion section ***(Figure 1, Page number: 14)***, with appropriate referencing and hope your further consideration.

Thank you for your kind consideration.

**RESPONSES TO REVIEWER 1**

1. **Reviewer’s comment:** I am satisfied by the revisions made to the paper.

***Authors’ response:*** The authors highly appreciate the positive feedback of the reviewer.

Thank you for your consideration.
